# Commentary: BMI is associated with sperm quality and sex hormones in men: a meta-analysis

**DOI:** 10.3389/fendo.2026.1788178

**Published:** 2026-03-12

**Authors:** Shanshan Wu, Cheng Zhang

**Affiliations:** Clinical Laboratory Department, The Affiliated Hospital of Liaoning University of Traditional Chinese Medicine, Shenyang, Liaoning, China

**Keywords:** BMI, fixed-effects model, luteinizing hormone, meta-analysis, random-effects model, sex hormones, sperm quality, total testosterone

## Introduction

We have read with great interest the article by Ji and colleagues, entitled “BMI is associated with sperm quality and sex hormones in men: a meta-analysis” (1). The authors provide valuable evidence indicating that elevated BMI is significantly associated with impaired semen quality and altered sex hormone levels in men (1). Although interpretation of these findings must consider potential heterogeneity arising from differences in participant sources, the consistent adverse effect of BMI across various populations strongly supports its inclusion as a routine component in the clinical evaluation of male fertility (1). A review of the literature identified possible data entry errors in Ji and colleagues’analysis (1), which may have led to inconsistencies in the reported results. In our re-analysis using corrected data, the subgroup comparison showed no statistically significant difference in luteinizing hormone (LH) concentrations between overweight and obese individuals (*P* = 0.25), in contrast to the original study, which reported a significant difference between these groups (1).

A critical assessment of the methodological concerns to subgroup analysis is imperative. Specifically, a clear discrepancy exists between the statistical approach described in Section 2.6 and its actual implementation in the study (1). The authors state that a random-effects model would be applied in cases of high heterogeneity (I² > 50% or P < 0.1) —conditions that were explicitly met in their results (*I*² = 98% for Figure 4A; *I*² = 96% for Figure 4D) (1). Despite this, the forest plots in Figures 4A, D indicate the use of a fixed-effects model (1). This model misapplication has produced erroneous statistically significant findings: first, regarding total testosterone (TT) concentration in the normal weight versus obesity comparison (Figure 4A), and second, regarding luteinizing hormone (LH) concentration between overweight and obese individuals (Figure 4D) (1). The present commentary seeks to correct this analytical error and present a revised analysis using the appropriate random-effects model.

## Statistical analysis

We utilized the same dataset and adhered to the same inclusion criteria as those specified by Ji et al. All statistical analyses were performed using RevMan software (version 5.3). For continuous variables, the mean difference (MD) was employed for outcomes measured with uniform units, whereas the standardized mean difference (SMD) along with a 95% confidence interval (CI) was applied for outcomes with differing units. Heterogeneity across studies was evaluated using the I² statistic, with values exceeding 25%, 50%, and 75% indicating low, moderate, and high heterogeneity, respectively. When I² was 50% or higher, sensitivity or subgroup analyses were performed, and a random-effects model was adopted. In cases where I² was below 50%, a fixed-effects model was used (2). Thus, the selection of the statistical model was determined based on the observed I² value. A p-value of less than 0.05 was regarded as statistically significant.

## Revised meta-analysis results

**Figure 1:**
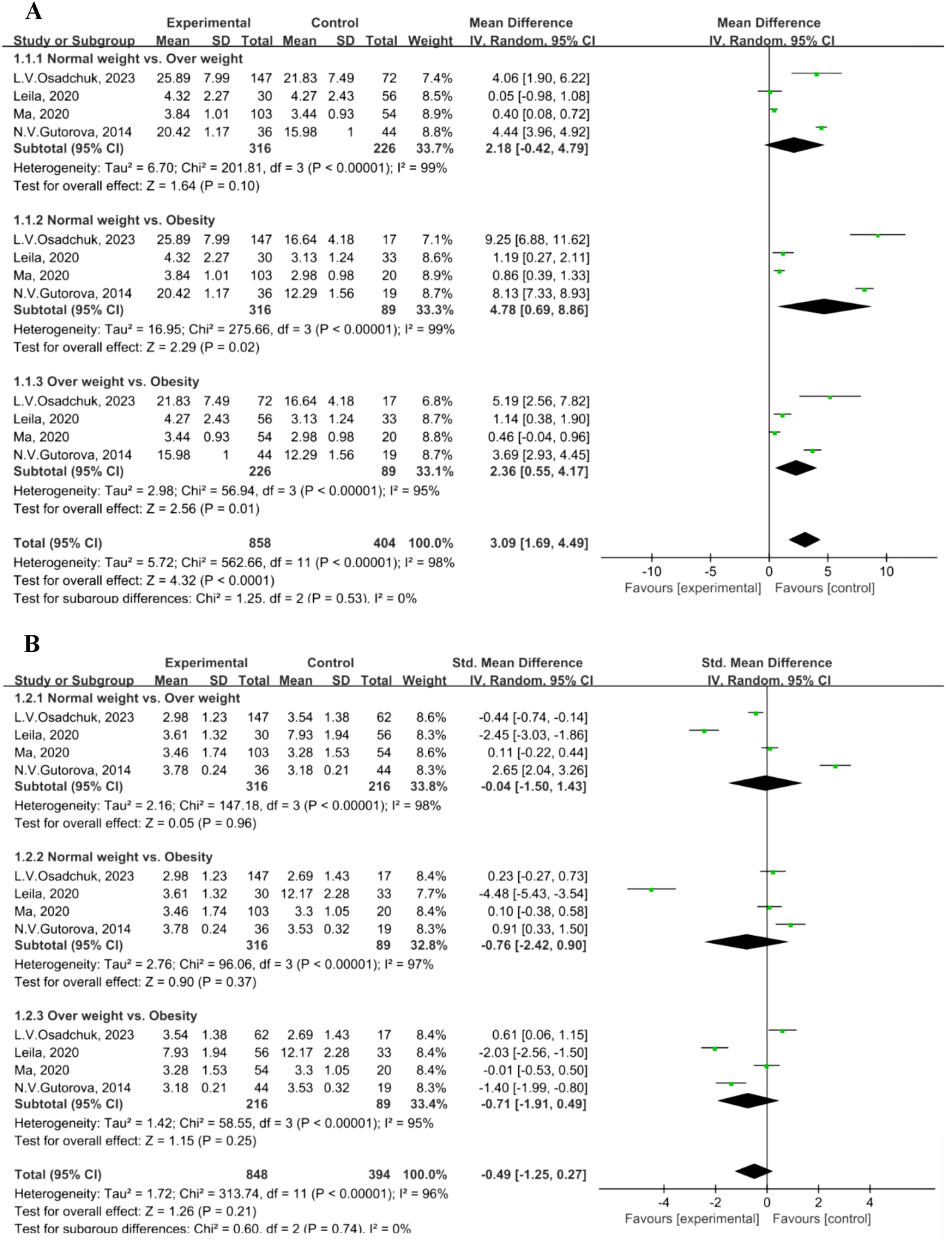
Forest plot of indicators. **(A)** Forest plot of total testosterone level. **(B)** Forest plot of luteinizing hormone level.

1. In Figure 4D (1), the standard deviation (SD) is 1.23 in the normal weight group of the study by L. V. Osadchuk 2023 (3), instead of 3.54.
2. A reanalysis of the BMI-stratified data (from four studies) revealed notable between-group heterogeneity in effect sizes (*I*² = 98% in **Figure 1A**; *I*² = 95% in **Figure 1B**). Consequently, a random-effects model was applied.
3. Subgroup analysis of TT concentration revealed statistically significant differences in the comparisons of normal weight versus obesity (MD = 4.78, 95% CI: 0.69–8.86, P = 0.02; **Figure 1A**) and overweight versus obesity (MD = 2.36, 95% CI: 0.55–4.17, P = 0.01; **Figure 1A**). In contrast, no significant difference was observed between the normal weight and overweight groups (MD = 2.18, 95% CI: -0.42–4.79, P = 0.10; **Figure 1A**). Normal weight: 18.5–24.9 kg/m², overweight: 25.0–29.9 kg/m², and obesity: ≥30.0 kg/m² (4).
4. No significant differences in LH concentration were found in the subgroup analyses: normal weight versus overweight (MD = -0.04, 95% CI: -1.50–1.43, *P* = 0.96); normal weight versus obesity (MD = -0.76, 95% CI: -2.42–0.90, *P* = 0.37); and overweight versus obesity (MD = -0.71, 95% CI: -1.91–0.49, *P* = 0.25) (**Figure 1B**).

## Discussion

This re-analysis was prompted by the identification of a data entry discrepancy and a critical methodological inconsistency in the original meta-analysis by Ji et al. (1). regarding the relationship between BMI and male reproductive hormones. The original study presents a valuable synthesis; however, we identified a critical methodological issue: its conclusions on TT and LH comparisons relied on an incorrect statistical model (specifically, the use of a fixed-effects model under high heterogeneity), which affects the interpretation of results and leads to potentially inaccurate conclusions (1).

The primary methodological concern pertains to the choice of the meta-analytic model, which is a fundamental decision point in evidence synthesis. Standard methodological guidance indicates that in the presence of significant statistical heterogeneity, a random-effects model is more appropriate than a fixed-effects model, as the former accounts for variability beyond sampling error among studies (5). The choice between these models should be explicitly justified, as careless application can lead to misleading inferences about treatment effects (6). Ji et al. explicitly stated that a random-effects model would be employed in cases of high heterogeneity (I² > 50%) (1). This condition was unequivocally met in their own analyses for TT and LH (I² = 98% and 96%, respectively). However, the forest plots presented demonstrate the application of a fixed-effects model, creating a discrepancy between the stated protocol and its execution. These results highlight a key methodological limitation: the application of a fixed-effects model in the presence of substantial heterogeneity artificially inflates the precision of the pooled estimate (as indicated by inappropriately narrow confidence intervals), which can lead to an overestimation of clinical significance (7). It is also crucial to note that while the I² statistic is a useful indicator, it should not be the sole criterion for deciding whether to pool studies; its interpretation must consider the precision and context of the included studies (8). Notably, the findings from our re-analysis do not change the principal conclusion reached by Ji et al.: an elevated BMI is independently linked to poorer sperm quality and disrupted sex hormone balance. As such, BMI should be incorporated as a key risk factor in male fertility evaluations.

In conclusion, while the overarching conclusion of Ji et al. remains supported, our re-analysis provides a necessary methodological correction and refinement. It demonstrates that the relationship between BMI and specific sex hormones may be less pronounced than initially suggested when appropriate statistical models are employed. This case emphasizes that the reliability of pooled evidence depends not only on the quality of the included data but also on strict adherence to pre-specified, statistically sound methodologies.
